# Unusual Degeneration of the Cervix

**Published:** 1916-04

**Authors:** 


**Unusual Degeneration of the Cervix.** Alfred Smith, reporting to the Irish Academy of Medicine, said that the patient, aged sixty, the mother of sixteen children, consulted him on account of a profuse slimy discharge. The menopause came on ten years ago. She enjoyed good health up to last August, when she noticed a slimy discharge from her vagina. There was neither hemorrhage nor pain nor offensive odor. On making a bimanual examination, the vaginal portion seemed to have disappeared, the margins being flush with the vaginal vault, the os being so dilated that the index and middle finger could easily be passed in. The impression conveyed was that there was no internal os, that the cavity of the uterus was greatly dilated; the mucous membrane felt like velvet pile. This slight palpation caused hemorrhage. On curettage, large quantities of brain-like matter came away, as in cancer.

The pathologist reported non-malignant. A simple panhysterectomy was performed. The total length of the uterus was 12 c.m., fundus 3.5 c.m., cervix 8.5 c.m., greatest width 6.7 c.m. The fundus of the uterus is normal, but the cervical portion is greatly thickened. On section, this thickening is seen to be due to the transformation of the normal muscle wall into a spongy mass infiltrated with mucoid material; this mucoid material is directly continuous with a large amount of mucus in the cavity of the cervix. This change affects the whole contour of the cervix, though it is more marked in the anterior and left wall. The remains of the true cervical wall are represented by a thin layer of fibro-muscular tissue. No evidence of malignancy. The specimen is in the nature of a channeled mucous polypus, but is remarkable in that it engages more or less uniformly the whole of the cervical wall.—(Dublin Jour. Med. Sci., Sept., 1915).—Med. Times, Dec.

